# Towards Volatile Organoselenium Compounds with Cost-Effective Synthesis

**DOI:** 10.3390/molecules25215212

**Published:** 2020-11-09

**Authors:** Jaroslav Charvot, Daniel Pokorný, Milan Klikar, Veronika Jelínková, Filip Bureš

**Affiliations:** 1 Institute of Organic Chemistry and Technology, Faculty of Chemical Technology, University of Pardubice, Studentská 573, 53210 Pardubice, Czech Republic; st41999@student.upce.cz (J.C.); st56842@student.upce.cz (D.P.); milan.klikar@upce.cz (M.K.); 2 The Institute of Technology and Business in České Budějovice, Okružní 517/10, 370 01 České Budějovice, Czech Republic; 24579@mail.vstecb.cz

**Keywords:** selenium, facile synthesis, volatility, thermal properties

## Abstract

The current portfolio of organoselenium compounds applicable as volatile precursors for atomic layer deposition can be denoted as very limited. Hence, we report herein facile and cost-effective preparation of two bis(trialkylstannyl)selenides as well as one selenole and three bis(trialkylsilyl)selenides. Their syntheses have been optimized to: (i) use readily available and inexpensive starting materials, (ii) involve operationally simple methodology (heating in a pressure vessel), (iii) use a minimum amount of additives and catalysts, and (iv) either exclude additional purification or involve only simple distillation. The chemical structure of prepared Se derivatives was confirmed by multinuclear NMR and GC/MS. Their fundamental thermal properties were investigated by differential scanning calorimetry (DSC) and TGA methods that revealed thermal stability within the range of 160–300 °C.

## 1. Introduction

**Scheme 1:**
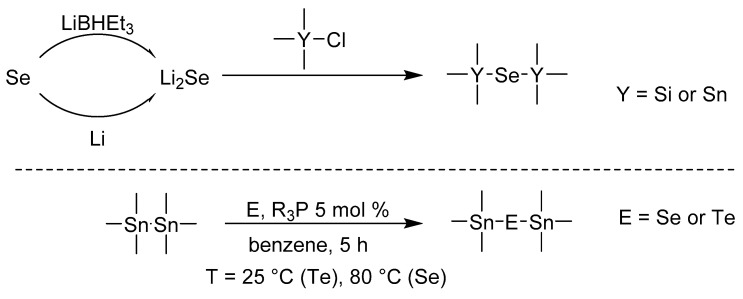
Known synthetic approaches towards (R_3_Y)_2_Se and (Me_3_Sn)_2_E compounds.

In addition to its indisputable essential and biological importance, selenium and its organo derivatives have also found important applications in materials science. For instance, chalcogenide forms metal selenides that belong to a group of TMDs (Transition Metal Dichalcogenides) with highly exploitable potential in many fields. Thin layers of metal sulfides, selenides, and tellurides have turned out to be very active in electrocatalysis; this topic was recently summarized by Lei [1]. Sb_2_Se_3_ is another example that showed interesting performance in optoelectronics [2]. Vanadium [3] or tungsten selenides [4] are well-known semiconducting materials for electronic technologies such as spintronics, oscillators, or memory storage devices. The successful application of the aforementioned selenides relies on their supramolecular arrangement, which is mostly affected by the synthetic method and deposition technique used. In this respect, Atomic Layer Deposition (ALD) is a burgeoning coating technology, which allows the manufacture of various thin films. The self-limiting nature of ALD provides a high compactness of nanolayer with exceptional coverage [5]. This holds true even for differently shaped substrates such as nanotubes [6], while ALD also allows a selective deposition over the particular substrate surface area [7]. ALD is a cyclic process composed of a few basic steps. The first one involves the reaction of the ALD precursor with free surface functional groups of the substrate in the reaction chamber. Subsequent evacuation of the chamber ensures the removal of precursor excess and possible by-products. After insertion of a second precursor, which is the third step, an atomic layer is deposited. The number of these cycles defines the thickness of the manufactured thin film, while the selection of the precursors allows the design of its composition. For instance, using water or oxygen leads to oxides, H_2_S to sulfides etc. However, the situation with metal precursors is complicated because such precursors need to be transported in a gas phase. Hence, the molecule used must be volatile enough and possess a good trade-off between thermal stability and chemical reactivity. In fact, there are only a few ALD precursors that deposit metal selenides. The previously used Et_2_Se (Et_2_Se_2_), which usually does not provide desired results, and toxic H_2_Se were mostly replaced with bis(trialkylsilyl)selenides [8]. In 2019, selenium dimethyldithiocarbamate, which reacts with tris(dimethylamino)antimony to form a Sb_2_Se_3_ nanolayer, was introduced by Sarkar et al. [9]. We have recently reported on a series of cyclic-silylselenides; in particular, the six-membered derivative bearing two selenium atoms provided very promising results during the depositing of MoSe_2_ by ALD [10]. Our further synthetic work [11] has identified bis(trialkylstanyl)selenides, structurally related to bis(trialkylsilyl)selenides, as an ALD selenium precursor with lowered air sensitivity. Indeed, (Me_3_Sn)_2_Se turned out to be a useful selenium source for MoSe_2_ nanolayers; however, the previous work lacked in-depth optimization of the synthesis. Easy and cost-effective preparation of the given organoselenium compound is another important aspect that must be considered when designing a new ALD precursor. Scheme 1 shows two reactions that in our opinion possess potential to be used for the preparation of selenium ALD precursors.

The most straightforward and efficient synthesis towards compounds of general formula (R_3_Y)_2_Se involves the in situ preparation of Li_2_Se, which subsequently reacts with trialkylsilyl chloride. Depending on the R-alkyl groups, the obtained yields vary from 43 to 90% [11,12,13,14,15,16,17]. Li_2_E is usually generated by the reaction of elemental selenium with either lithium or superhydride. Although the first approach is time-consuming and provides generally lower yields, the latter is fast but uses expensive LiBHEt_3_. Another interesting synthesis was reported by Tanaka et al. [18]. They used catalytic R_3_P oxidation by elemental Se or Te affording R_3_P = E. These agents are capable of inserting chalcogen into Sn–Sn (and Pb–Pb) bond (Scheme 1). Depending on the used catalyst, the yields reported for (Me_3_Sn)_2_Se were up to 46% (using Ph_3_P).

## 2. Materials and Methods

### 2.1. Synthesis of Selenium Precursors

All reactions were performed under argon using the Schlenk technique. The used solvents were water- and oxygen-free. Organic selenides are generally toxic; thus, all used glassware was treated with sodium hypochlorite after experiments. The reactions were carried out in Ace pressure tubes (Merck, Kenilworth, NJ, USA). A standard oil bath and a magnetic stirrer were used to accomplish the heating and stirring of the reaction mixtures. ^1^H, ^13^C, ^29^Si, and ^77^Se NMR spectra were recorded with Bruker AVANCE 400 and Bruker Ascend^TM^ 500 instruments equipped with a cryo-probe at 25 °C. Chemical shifts are reported in ppm relative to the signal of Me_4_Si. The MS spectra were measured with a GC/EI–MS configuration including gas chromatography Agilent Technologies 6890N (HP–5MS, 30 m column, I.D. 0.25 mm, film 0.25 µm) equipped with a mass detector Network MS detector 5973 (EI 70 eV, range 33–550 Da).

#### 2.1.1. Bis(Trimethylstannyl)Selenide—(Me_3_Sn)_2_Se

Me_6_Sn_2_ (5.0 g; 15.26 mmol) and fine selenium powder (1.4 g; 17.73 mmol) were placed into a pressure vessel and heated to 180 °C for 5 h. After cooling to room temperature, the selenium excess was filtered off to give 6.1 g (98%) of (Me_3_Sn)_2_Se as a yellow liquid.

^1^H–NMR (500 MHz, 25 °C, CDCl_3_): *δ* = 0.51 (s, 18H, CH_3_) ppm. ^13^C–NMR APT (125 MHz, 25 °C, CDCl_3_): *δ* = −1.86 ppm. ^119^Sn–NMR (186 MHz, 25 °C, CDCl_3_): *δ* = 50.06 ppm. ^77^Se–NMR (95 MHz, 25 °C, CDCl_3_): *δ* = −550.76 ppm. EI–MS: *m/z* = 408 (10, M^+^), 391 (40), 165 (100), 135.

#### 2.1.2. Bis(Tributylstannyl)Selenide—(Bu_3_Sn)_2_Se

Bu_6_Sn_2_ (5.0 g; 4.35 mL; 8.62 mmol) and fine selenium powder (0.82 g, 10.34 mmol) were placed into the pressure vessel and heated to 180 °C for 12 h. After cooling to room temperature, the selenium excess was filtered off to give 5.6 g (98%) of (Bu_3_Sn)_2_Se as a yellow oil.

Bu_3_SnH (3.7 g; 3.42 mL; 12.71 mmol) and selenium powder (0.6 g; 7.6 mmol) were placed into a pressure vessel and heated to 180 °C for 12 h. After cooling to room temperature, the selenium excess was filtered off to give 4 g (98%) of (Bu_3_Sn)_2_Se as a yellow oil.

^1^H–NMR (500 MHz, 25 °C, CDCl_3_): *δ* = 0.91 (t, *J* = 7.5 Hz, 18H, CH_3_), 1.12 (t, *J* = 8.2 Hz, 12H, CH_2_), 1.30–1.38 (m, 12H, CH_2_), 1.53–1.60 (m, 12H, CH_2_) ppm. ^13^C–NMR APT (125 MHz, 25 °C, CDCl_3_): *δ* = 13.61, 15.44, 27.08, 29.00 ppm. ^119^Sn–NMR (186 MHz, 25 °C, CDCl_3_): *δ* = 57.14 ppm. ^77^Se–NMR (95 MHz, 25 °C, CDCl_3_): *δ* = −651.72 ppm. EI–MS: *m/z* = 545 (20), 317, 291 (90), 235 (50), 179 (100).

#### 2.1.3. Tri*iso*propylsilylselenole—*i*Pr_3_Si–SeH

*i*Pr_3_Si–H (3.86 g; 5.0 mL; 24.4 mmol), Se powder (2.3 g; 29.29 mmol), and Ph_3_P (50 mg; 0.19 mmol) were placed into a pressure vessel and the reaction mixture was heated to 250 °C for 48 h. After cooling to room temperature, the selenium excess was filtered off. The residue was purified by vacuum distillation at 80–85 °C (5 torr) to give 4.68 g (81%) of *i*Pr_3_Si–SeH as an orange liquid.

^1^H–NMR (400 MHz, 25 °C, C_6_D_6_): *δ* = −2.81 (s, 1H, SeH), 0.98–1.04 (m, 21H, CH, CH_3_) ppm. ^13^C–NMR APT (100 MHz, 25 °C, C_6_D_6_): *δ* = 13.96, 18.72 ppm. ^29^Si–NMR (99 MHz, 25 °C, C_6_D_6_): *δ* = 32.22 ppm. ^77^Se–NMR (95 MHz, 25 °C, C_6_D_6_, gated): *δ* = −420.7 (d, *J* = 47.17 Hz) ppm. EI–MS: *m/z* = 238 (20, M^+^) 195 (100), 153 (60), 125 (65), 59 (15).

#### 2.1.4. Bis(Dimethylphenylsilyl)Selenide—PhMe_2_Si–Se–SiMe_2_Ph

Selenium powder (0.5 g; 6.33 mmol), PhMe_2_Si–H (2.6 g; 3.0 mL; 19 mmol), and Ph_3_P (10 mg) were placed into a pressure vessel and the reaction mixture was heated to 250 °C for 48 h. Vacuum distillation of the crude product at 160–165 °C (2 torr) gave 1.0 g (43%) of a colorless oil.

^1^H–NMR (400 MHz, 25 °C, C_6_D_6_): *δ* = 0.45 (s, 12H, CH_3_), 7.14–7.16 (m, 6, Ph), 7.50–7.52 (m, 4H, Ph) ppm. ^13^C–NMR APT (100 MHz, 25 °C, C_6_D_6_): *δ* = 127.93, 129.66, 133.94, 138.44 ppm. ^29^Si–NMR (99 MHz, 25 °C, C_6_D_6_): *δ* = 5.44 ppm. ^77^Se–NMR (95 MHz, 25 °C, C_6_D_6_): *δ* = −341.15 ppm. EI–MS: *m/z* = 350 (10, M^+^), 257 (10), 197 (20), 135 (100).

#### 2.1.5. Bis(Triethylsilyl)Selenide—Et_3_Si–Se–SiEt_3_

Selenium powder (0.5 g; 6.33 mmol), Et_3_Si–H (1.5 g; 2.0 mL; 12.66 mmol), Ph_3_Si–H (30 mg), and Ph_3_P (10 mg) were placed into a pressure vessel and the reaction mixture was heated to 250 °C for 48 h. After cooling to room temperature, the mixture was filtered and purified by vacuum distillation at 120–125 °C (3 torr) to give 0.7 g (35%) of an orange liquid.

^1^H–NMR (500 MHz, 25 °C, CDCl_3_): *δ* = 0.81 (q, *J* = 8.0 Hz, 12H, CH2), 1.01 (t, *J* = 8.0 Hz, 18H, CH_3_) ppm. ^13^C–NMR APT (125 MHz, 25 °C, CDCl_3_): *δ* = 7.70, 8.16 ppm. ^29^Si–NMR (99 MHz, 25 °C, CDCl_3_): *δ* = 23.41 ppm. ^77^Se–NMR (95 MHz, 25°C, CDCl_3_): *δ* = −485.70 ppm. EI–MS: *m/z* = 310 (20, M^+^), 281 (100), 253 (60), 115 (70), 87, 59.

#### 2.1.6. Tri*iso*propylsilyl–Trimethylsilylselenide—*i*Pr_3_Si–Se–SiMe_3_

*i*Pr_3_Si–H (3.86 g; 5.0 mL; 24.4 mmol), Se powder (2.3 g; 29.29 mmol), and Ph_3_P (50 mg; 0.19 mmol) were placed into a pressure vessel and the reaction mixture was heated to 250 °C for 48 h. After cooling to room temperature, hexane (30 mL) and EtN*i*Pr_2_ (3.15 g; 4.26 mL, 24.4 mmol) were added. The mixture was cooled to 0 °C and Me_3_SiCl (2.64 g; 3.58 mL; 24.4 mmol) was added dropwise. After stirring for 12 h at 25 °C, the mixture was filtered, and hexane was evaporated in vacuo. The residue was purified by vacuum distillation at 100–105 °C (2 torr) to give 3.6 g (50%) of a yellow liquid.

Hexane (30 mL), *i*Pr_3_Si–SeH (1.22 g; 5.14 mmol) and EtN*i*Pr_2_ (0.67 g; 0.9 mL; 5.14 mmol) were placed into a Schlenk flask and the reaction mixture was cooled to 0 °C. Me_3_SiCl (0.56 g; 0.65 mL; 5.14 mmol) was added dropwise and the mixture was stirred for 12 h at 25 °C. Upon filtration and vacuum distillation at 100–105 °C (2 torr), 1.12 g (70%) of a yellow liquid was obtained.

^1^H–NMR (400 MHz, 25 °C, C_6_D_6_): *δ* = 0.40 (s, 9H, CH_3_), 1.01 (s, 3H, CH), 1.11–1.15 (m, 18H, CH_3_) ppm. ^13^C–NMR APT (100 MHz, 25 °C, C_6_D_6_): *δ* = 4.97, 14.67, 19.09 ppm. ^29^Si–NMR (99 MHz, 25 °C, C_6_D_6_): *δ* = 10.41, 30.24 ppm. ^77^Se–NMR (95 MHz, 25 °C, C_6_D_6_): *δ* = −447.10 ppm. EI–MS: *m/z* = 310 (10, M^+^), 267 (95), 224 (45), 183 (20), 129 (20), 101 (25), 73 (100).

### 2.2. DSC and TGA Measurements

Differential scanning calorimetry (DSC) was carried out with a Mettler–Toledo STARe System DSC 2/700 equipped with FRS 6 ceramic sensor and cooling system HUBER TC100–MT RC 23. Thermal behavior of the target compounds was measured in open aluminous crucibles under N_2_ inert atmosphere. DSC curves were determined with a scanning rate of 3–5 °C/min within the range −70–+400 °C. Thermogravimetric analysis (TGA) was carried out using a Mettler–Toledo STARe System TGA 2 equipped with a horizontal furnace LF (400 W, 1100 °C), balance XP5 (resolution 1 μg) and cooling system HUBER Minichiller 600.

## 3. Results and Discussion

### 3.1. Straightforwad Reaction Pathway to Bis(Trialkylstannyl)Selenides

**Scheme 2:**
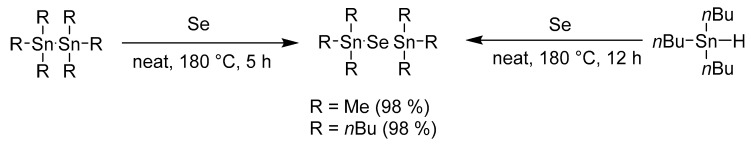
Tuned synthesis of (R_3_Sn)_2_Se at high temperature.

The work of Tanaka et al. [18] reported that Me_6_Sn_2_ reacts sluggishly with elemental tellurium in the absence of a phosphine. This observation prompted us to investigate this un-catalyzed reaction in more detail. This goal was indeed achieved by simply tuning the reaction conditions and the compounds of general formula (R_3_Sn)_2_Se were prepared in a very straightforward manner without any catalyst (Scheme 2). The reaction carried out at a significantly raised temperature to 180 °C (using a pressure vessel) afforded (Me_3_Sn)_2_Se and (Bu_3_Sn)_2_Se in almost quantitative yields and no additional purification was required. It should also be highlighted that the starting Me_6_Sn_2_ and Bu_6_Sn_2_ are readily available and inexpensive compounds. On the contrary, a similar experiment with Me_6_Si_2_ with(out) Ph_3_P did not provide any product.

Triethyltinhydride has been reported as another starting material used for the synthesis of (Et_3_Sn)_2_Se [19]. However, its low commercial availability prompted us to attempt a reaction between inexpensive and readily available Bu_3_SnH and elemental selenium under similar high-temperature conditions (Scheme 2). No matter what amount of tributyltinhydride was used, bis(tributyltin)selenide was always exclusively isolated in almost quantitative yield.

### 3.2. Elaboration with Silylselenides

**Scheme 3:**
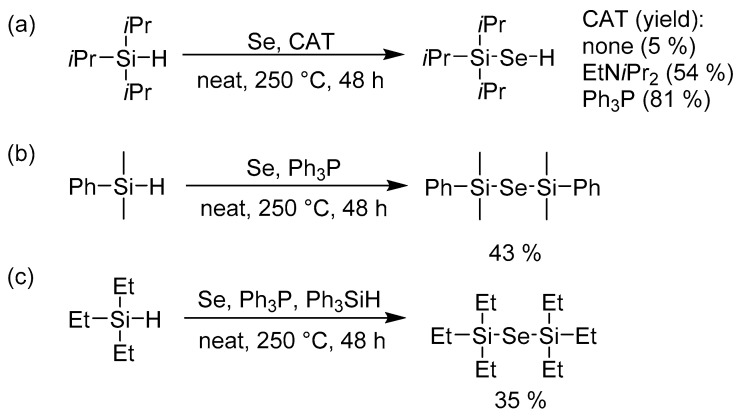
Towards trialkylsilylselanole and bis(trialkyl/aryl)silylselenides using tri*iso*propyl- (**a**), dimethylphenyl- (**b**), and triethylsilane (**c**).

Encouraged by the easy and high-yielding reaction with Bu_3_SnH (Scheme 2), we were also curious about a similar reaction with R_3_SiH. Such a reaction using one equivalent of trialkylsilane provides organoselenium compounds of general formula R_3_Si–SeH as reported for Et_3_Si–SeH [20] and cHex_3_Si–SeH [21]. Hence, we attempted the synthesis of Et_3_Si–SeH [20], but even after one week at different temperatures (120, 180, 250 °C) the attained yields did not exceed 1% (as monitored by GC/MS). Using *i*Pr_3_Si–H as a starting silane afforded 5% of *i*Pr_3_Si–SeH. Further elaboration revealed that the yield could be significantly improved using a catalytic amount of Lewis bases such as ethyldi*iso*propylamine or triphenylphosphane (Scheme 3a). Despite working with large excess of *i*Pr_3_Si–H, we observed exclusive formation of *i*Pr_3_Si–SeH and only traces of (*i*Pr_3_Si)_2_Se were detected on GC/MS. However, when replacing the starting tri*iso*propylsilane by dimethylphenylsilane, PhMe_2_Si–SeH has been identified as a minor product even if 1 equivalent of PhMe_2_Si–H was used. PhMe_2_Si–Se–SiMe_2_Ph was isolated in 43% yield when using 3 equivalents of PhMe_2_Si–H (Scheme 3b).

The initially unsuccessful preparation of Et_3_Si–SeH or well-established ALD precursor (Et_3_Si)_2_Se using the aforementioned methodology proved to be tricky. However, we further focused our attention to transfer of alkyl/aryl chains, well known for 14 group of elements. Hence, the reaction of selenium with 2 equivalents of Et_3_Si–H, Ph_3_P, and catalytic amount of Ph_3_Si–H provided desired (Et_3_Si)_2_Se in 35% yield (Scheme 3c). Only traces of Et_3_Si–SeH were detected by GC/MS.

**Scheme 4:**
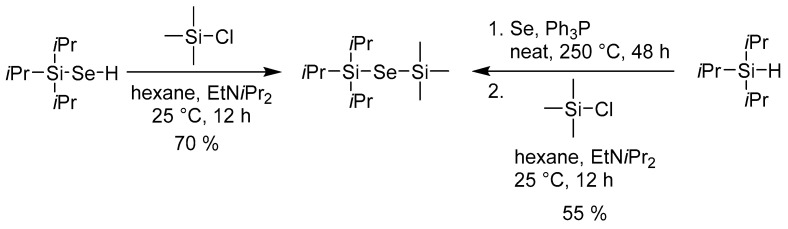
Preparation of unsymmetrical bis(trialkylsilyl)selenide.

In ALD, metal halides (X) are often used as the counter precursors. In this respect, selenoles R_3_SiSeH seem to be less useful as their joint reaction produces unfavorable and corrosive HX as a by-product. Hence, our further synthetic modifications were focused on overcoming this problem. Scheme 4 shows very simple replacement of the hydrogen (Se–H) by trimethylsilyl group by treating *i*Pr_3_Si–SeH with Me_3_SiCl in the presence of a base. This reaction could be performed either with isolated or in situ generated *i*Pr_3_Si–SeH and allows facile formation of unsymmetrical bis(trialkylsilyl)selenides, which is in contrast to previous reports [8,11].

The structure of the prepared organoselenium materials was determined by heteronuclear NMR spectroscopy and GC/MS analyses; the spectra are found in the Supporting Information files (Figures S1–S15). A very low chemical shift (−2.81 ppm) was observed for Se–H proton of *i*Pr_3_Si–SeH (Figure S1). ^77^Se–NMR gated spectrum of this compound also showed typical ^77^Se–^1^H coupling with *J* = 47.17 Hz (Figure S4). The recorded chemical shifts in ^77^Se–NMR vary from −447.1 ppm (*i*Pr_3_Si–Se–SiMe_3_) to −341.15 ppm (PhMe_2_Si–Se–SiMe_2_Ph). ^29^Si–NMR spectra revealed chemical shifts between 5.44 ppm (PhMe_2_Si–Se–SiMe_2_Ph) and 32.22 ppm (*i*Pr_3_Si–SeH). As expected, the GC/MS records always feature M^+^ as well as R_3_Y^+^ ions with a typical isotope layout for selenium accompanied by further fragmentation (Figures S13–S15).

### 3.3. Thermal Properties

**Figure 1:**
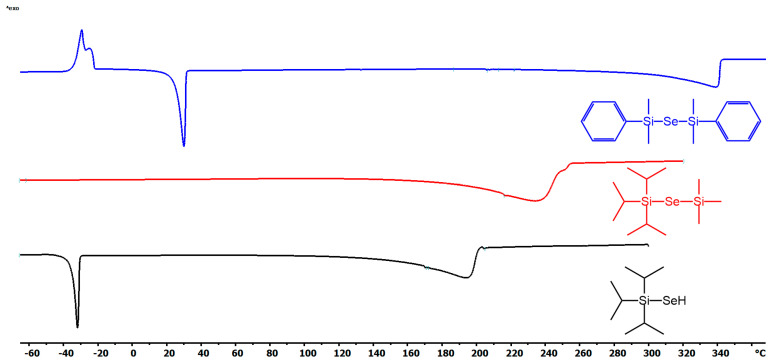
DSC records for newly prepared organoselenium compounds.

**Figure 2:**
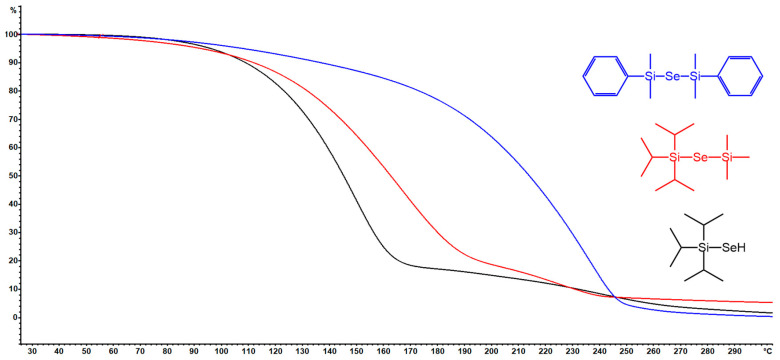
TGA curves for newly prepared organoselenium compounds.

Thermal stability and volatility are crucial properties of ALD precursors. The thermal properties of new organoselanoles and selenides (*i*Pr_3_Si–SeH, *i*Pr_3_Si–Se–SiMe_3_and PhMe_2_Si–Se–SiMe_2_Ph) were studied by DSC and TG analyses (see Figures S16–S21 in the Supporting Information for particular thermograms). For thermograms of remaining organoselanes, see our previous report [11]. The DSC curves shown in Figure 1 reveal melting/crystallization at −30/25 °C for *i*Pr_3_Si–SeH and PhMe_2_Si–Se–SiMe_2_Ph, while vigorous evaporation takes place mostly within the range of 120–200 (*i*Pr_3_Si–SeH), 280–340 (PhMe_2_Si–Se–SiMe_2_Ph), and 180–240 °C (*i*Pr_3_Si–Se–SiMe_3_). PhMe_2_Si–Se–SiMe_2_Ph derivative also showed cold crystallization at around −30 °C. Although the endothermic peaks of evaporation for PhMe_2_Si–Se–SiMe_2_Ph is sharp, another (probably exothermic) process accompanies the evaporation of the remaining two precursors. This is corroborated by the performed TG analyses shown in Figure 2. PhMe_2_Si–Se–SiMe_2_Ph showed gradual evaporation, while *i*Pr_3_Si–SeH and PhMe_2_Si–Se–SiMe_3_ underwent some additional unidentified thermal processes at around 160 and 190 °C. The mass residues are below 5%. Hence, at ambient pressure the prepared precursors are thermally stable up to 160 (*i*Pr_3_Si–SeH), 190 (*i*Pr_3_Si–Se–SiMe_3_), 200 (Me_3_Sn)_2_Se, (Bu_3_Sn)_2_Se, and (Et_3_Si)_2_Se), and 300 °C (PhMe_2_Si–Se–SiMe_2_Ph).

## 4. Conclusions

In conclusion, we have successfully optimized the synthesis of bis(trimethyl/butylstannyl)selenides. The synthesis is very straightforward and involves simple heating of the starting (Me_3_Sn)_2_Se, (Bu_3_Sn)_2_Se or Bu_3_SnH with selenium powder. Both products were isolated directly without the need for further purification and in almost quantitative yields. Moreover, all starting materials are readily available and inexpensive, which makes the developed synthesis cost-effective and suitable for large production (as generally required by the ALD).

Further attempts were focused on the synthesis of tri*iso*propylselenole, which was prepared in a similar easy way employing triphenylphosphane as a catalyst. Bis(dimethylphenylsilyl)selenide and bis(triethylsilyl)selenide were prepared analogously. We have further demonstrated that the Se–H bond in tri*iso*propylselenole can be modified, affording the first unsymmetrical (tri*iso*propylsilyl)(trimethylsilyl)selenide.

An investigation of the thermal properties with DSC and TGA revealed that thermal stability and volatility of all prepared derivatives are sufficiently high to be applied as Se precursors for the ALD.

## Supplementary Materials

The following are available online, Figure S1: ^1^H NMR (400 MHz, 25 °C, C_6_D_6_) spectrum of *i*Pr_3_Si–SeH, Figure S2: ^13^C–NMR (125 MHz, 25 °C, C_6_D_6_) spectrum of *i*Pr_3_Si–SeH, Figure S3: ^29^Si–NMR (99 MHz, 25 °C, C_6_D_6_) spectrum of *i*Pr_3_Si–SeH, Figure S4: ^77^Se–NMR (95 MHz, 25 °C, C_6_D_6_, gated) spectrum of *i*Pr_3_Si–SeH (detail around 420 ppm as an inset), Figure S5: ^1^H–NMR (400 MHz, 25 °C, C_6_D_6_) spectrum of PhMe_2_Si–Se–SiMe_2_Ph, Figure S6: ^13^C–NMR (125 MHz, 25 °C, C_6_D_6_) spectrum of PhMe_2_Si–Se–SiMe_2_Ph, Figure S7: ^29^Si–NMR (99 MHz, 25 °C, C_6_D_6_) spectrum of PhMe_2_Si–Se–SiMe_2_Ph, Figure S8: ^77^Se–NMR (95 MHz, 25 °C, C_6_D_6_) spectrum of PhMe_2_Si–Se–SiMe_2_Ph, Figure S9: ^1^H NMR (400 MHz, 25 °C, C_6_D_6_) spectrum of *i*Pr_3_Si–Se–SiMe_3_, Figure S10: ^13^C–NMR (125 MHz, 25 °C, C_6_D_6_) spectrum of *i*Pr_3_Si–Se–SiMe_3_, Figure S11: ^29^Si–NMR (99 MHz, 25 °C, C_6_D_6_) spectrum of *i*Pr_3_Si–Se–SiMe_3_, Figure S12: ^77^Se–NMR (95 MHz, 25 °C, C_6_D_6_) spectrum of *i*Pr_3_Si–Se–SiMe_3_, Figure S13: GC/MS record of *i*Pr_3_Si–SeH, Figure S14: GC/MS record of PhMe_2_Si–Se–SiMe_2_Ph, Figure S15: GC/MS record of iPr_3_Si–Se–SiMe_3_, Figure S16: DSC curve of *i*Pr_3_Si–SeH, Figure S17: DSC curve of PhMe_2_Si–Se–SiMe_2_Ph, Figure S18: DSC curve of *i*Pr_3_Si–Se–SiMe_3_, Figure S19: TGA curve of *i*Pr_3_Si–she, Figure S20: TGA curve of PhMe_2_Si–Se–SiMe_2_Ph, Figure S21: TGA curve of *i*Pr_3_Si–Se–SiMe_3_.
