# Supplementary material for: Towards Volatile Organoselenium Compounds with Cost-Effective Synthesis
Source: Molecules. 2020 Nov 9;25(21):5212. doi: 10.3390/molecules25215212 (PMC7664867; doi:10.3390/molecules25215212)
Supplement: Supplementary file 1 [file molecules-25-05212-s001.pdf]

molecules

Supplementary File

Towards Volatile Organoselenium Compounds with Cost-Effective Synthesis

Jaroslav Charvot [1], Daniel Pokorný [1], Milan Klikar [1], Veronika Jelínková [2] and Filip Bureš [1,2,*]

[1] Institute of Organic Chemistry and Technology, Faculty of Chemical Technology, University of Pardubice, Studentská 573, 53210 Pardubice, Czech Republic; st41999@student.upce.cz (J.C.); st56842@student.upce.cz (D.P.); milan.klikar@upce.cz (M.K.)

[2] The Institute of Technology and Business in České Budějovice, Okružní 517/10, 370 01 České Budějovice, Czech Republic; 24579@mail.vstecb.cz

* Correspondence: filip.bures@upce.cz; Tel.: +420-46-603-7099

Received: 25 September 2020; Accepted: 6 November 2020; Published: date

1. NMR Spectra of *i*Pr$_3$Si-SeH

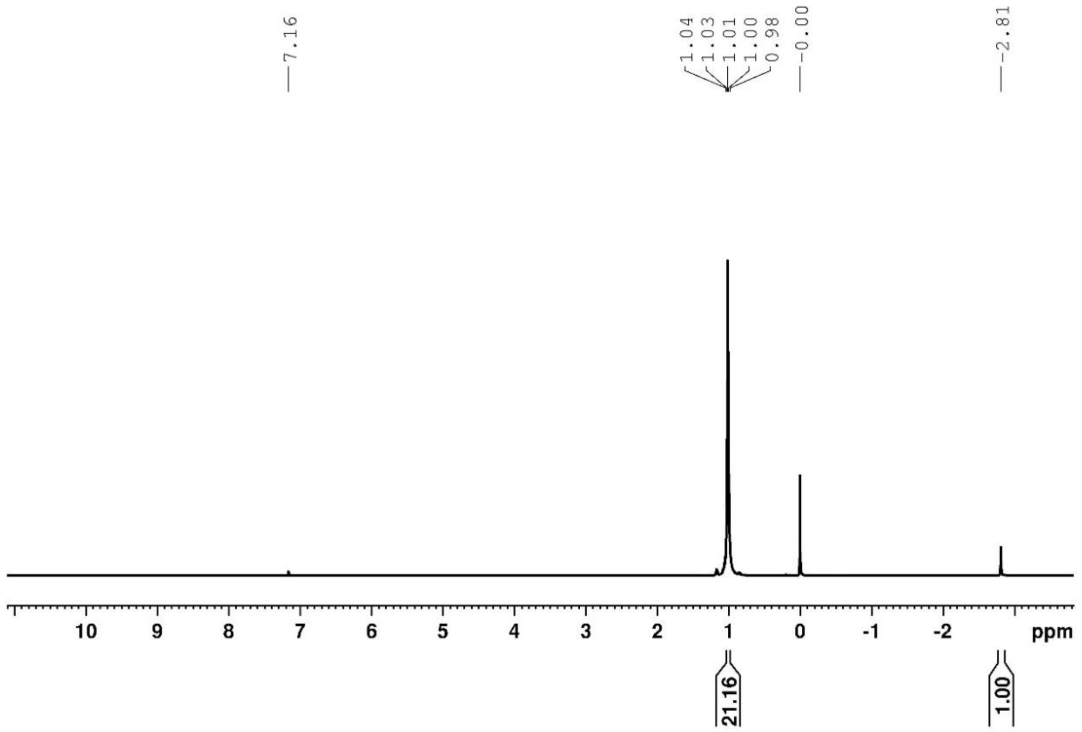

Figure S1. ^{1}H NMR (400 MHz, 25 °C, C_6D_6) spectrum of *i*Pr$_3$Si-SeH.

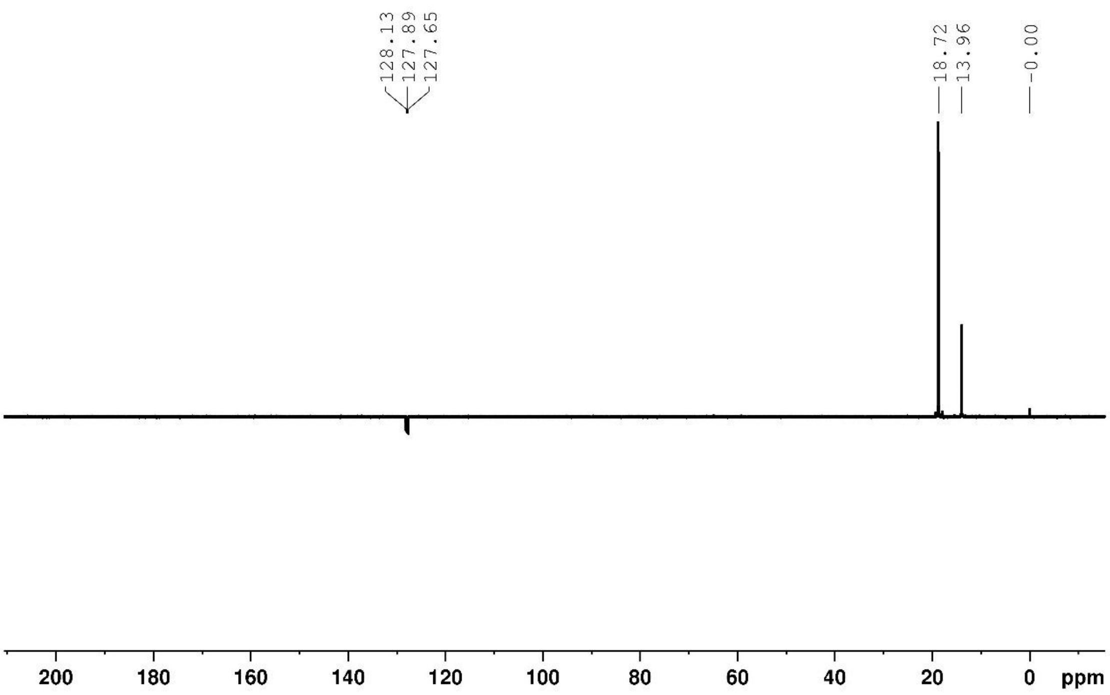

Figure S2. ^{13}C-NMR (125 MHz, 25 °C, C_6D_6) spectrum of *i*Pr_3Si-SeH.

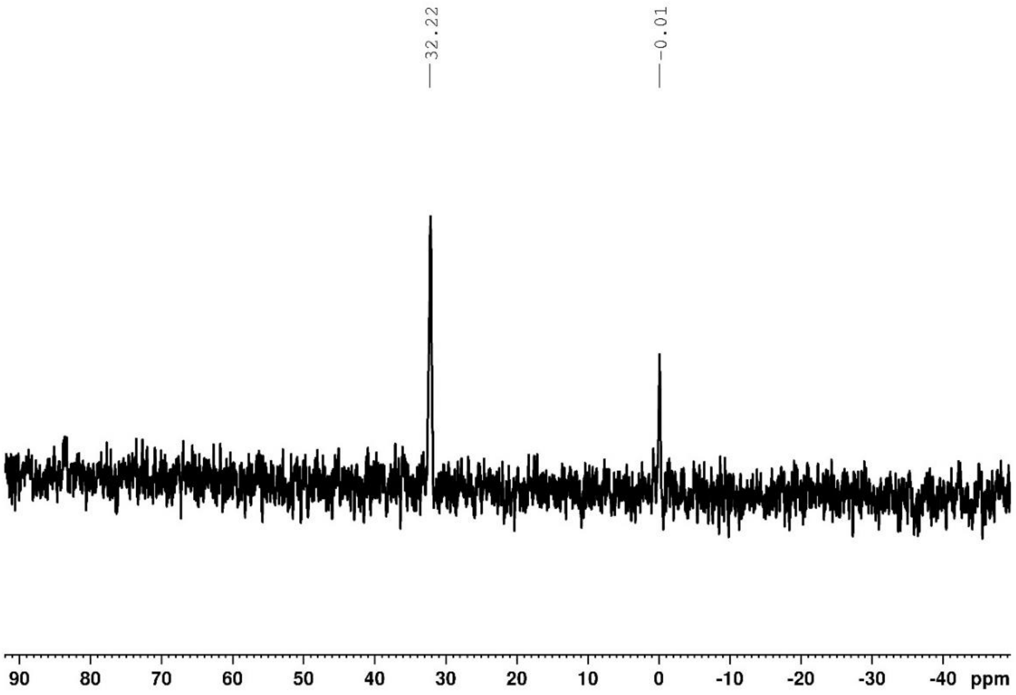

Figure S3. ^{29}Si-NMR (99 MHz, 25 °C, C_6D_6) spectrum of *i*Pr_3Si-SeH.

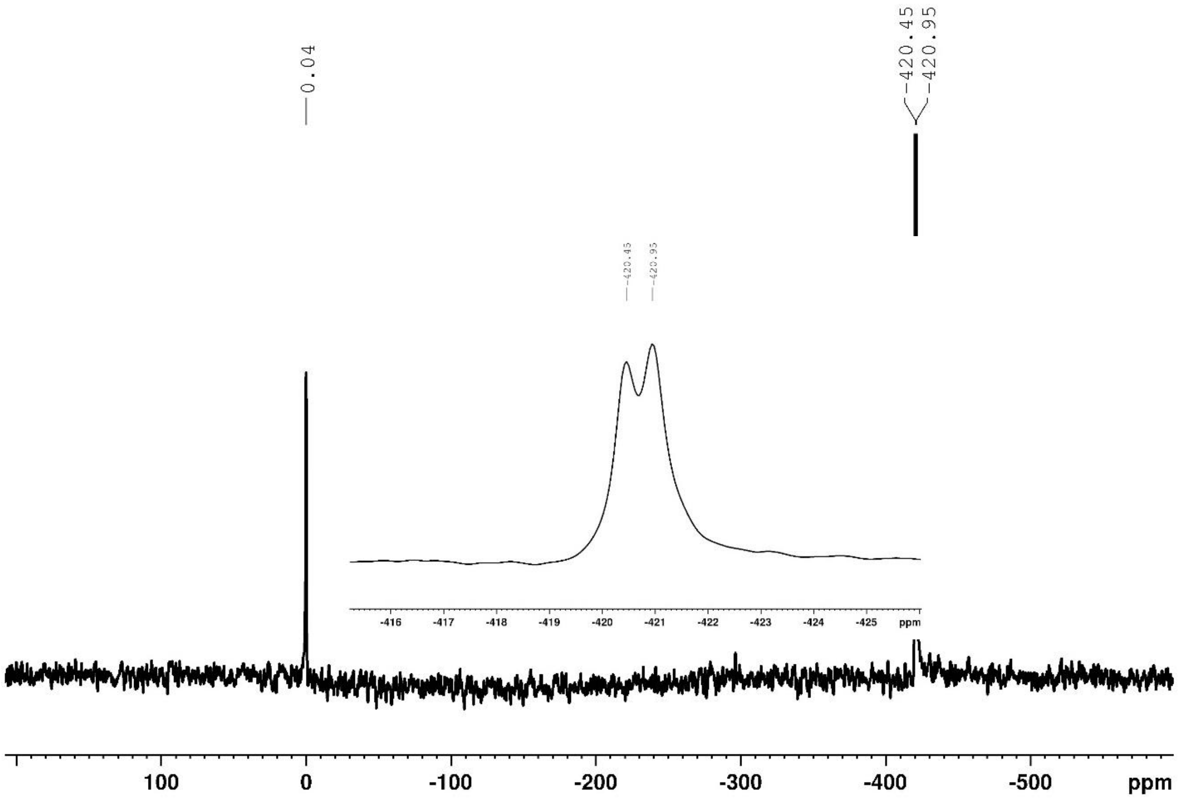

Figure S4. ^{77}Se-NMR (95 MHz, 25 °C, C_6D_6, gated) spectrum of *i*Pr_3Si-SeH (detail around 420 ppm as an inset).

2. NMR Spectra of $PhMe_2Si$-Se-$SiMe_2Ph$

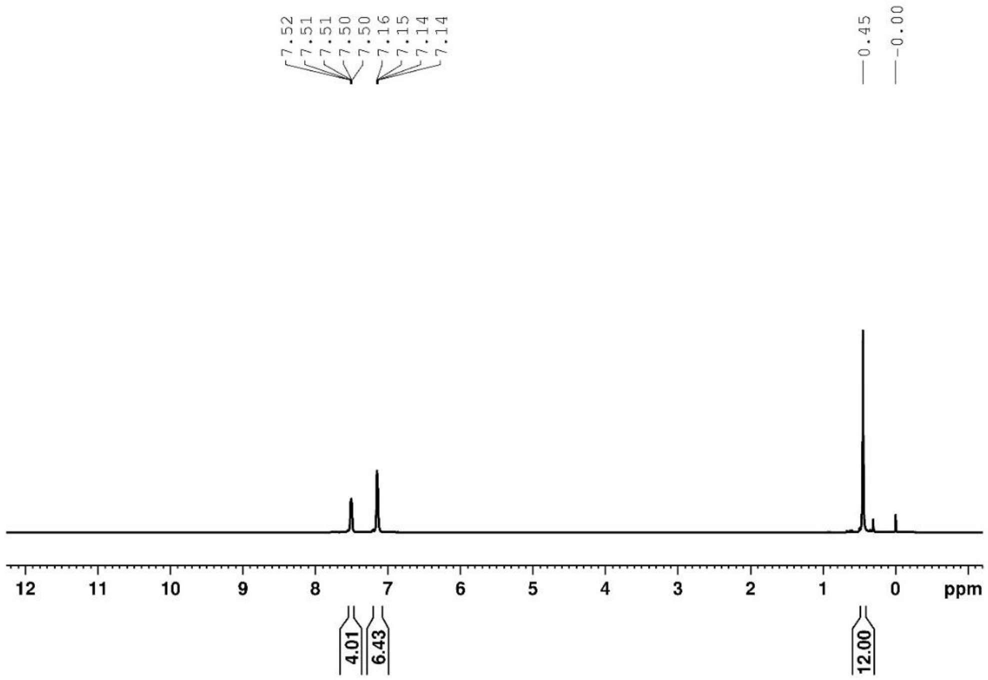

Figure S5. ^{1}H-NMR (400 MHz, 25 °C, C_6D_6) spectrum of $PhMe_2Si$-Se-$SiMe_2Ph$.

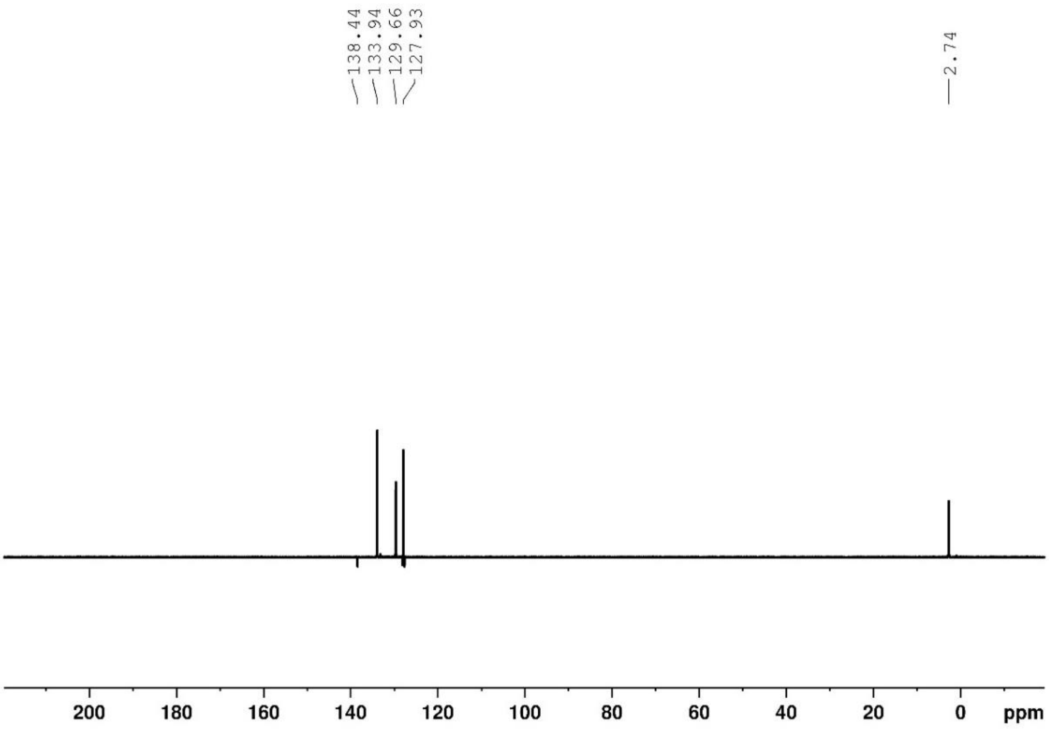

Figure S6. ^{13}C-NMR (125 MHz, 25 °C, C_6D_6) spectrum of $PhMe_2Si$-Se-$SiMe_2Ph$.

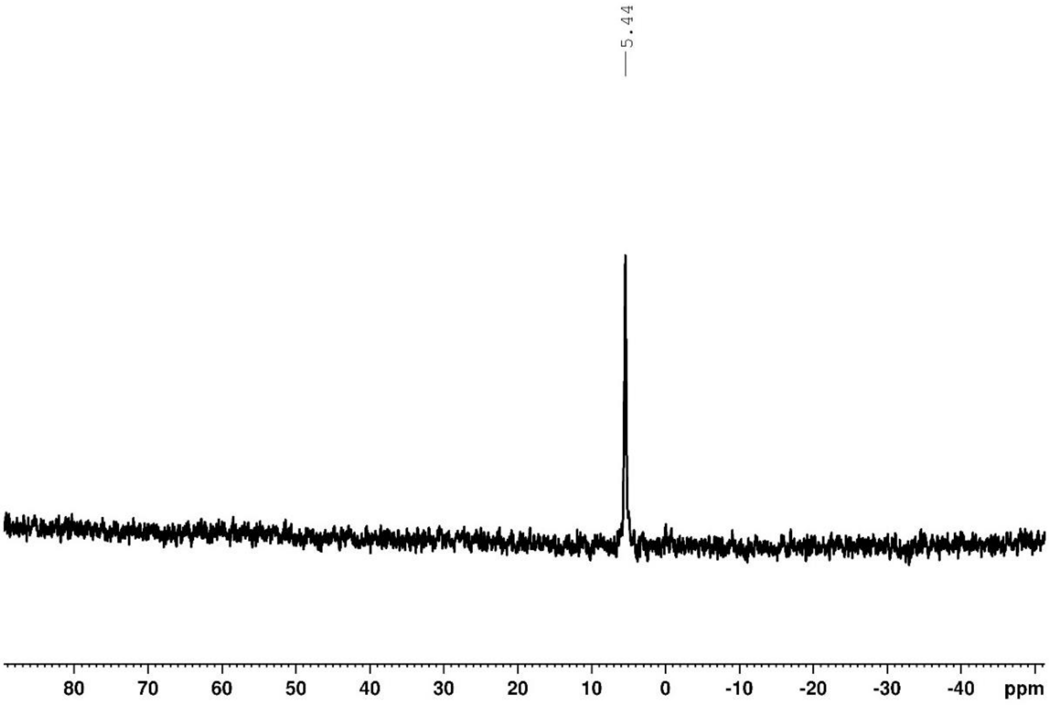

Figure S7. ^{29}Si-NMR (99 MHz, 25 °C, C_6D_6) spectrum of $PhMe_2Si$-Se-$SiMe_2Ph$.

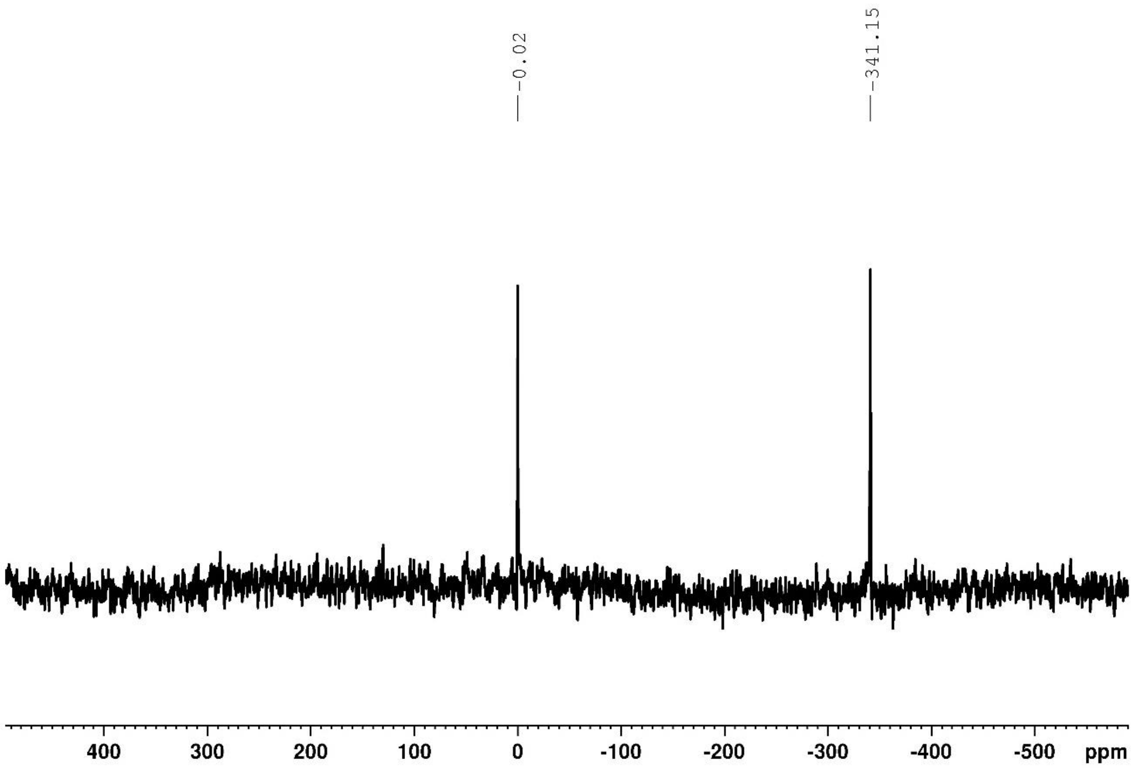

Figure S8. ^{77}Se-NMR (95 MHz, 25 °C, C_6D_6) spectrum of $PhMe_2Si$-Se-$SiMe_2Ph$.

3. NMR Spectra of *i*Pr_3Si-Se-$SiMe_3$

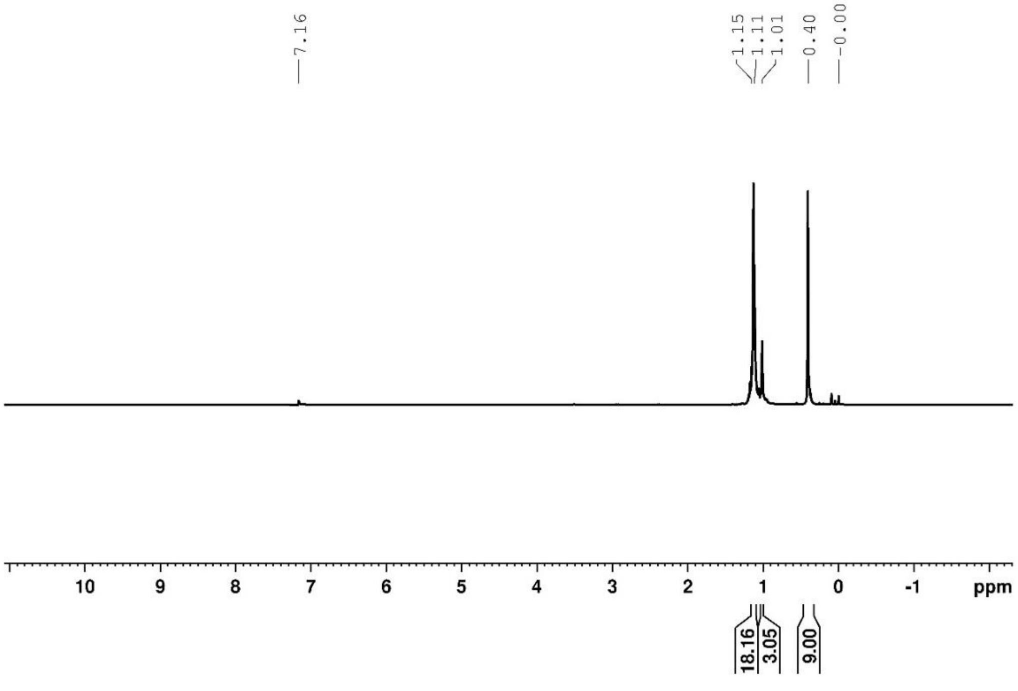

Figure S9. ^{1}H NMR (400 MHz, 25 °C, C_6D_6) spectrum of *i*Pr_3Si-Se-$SiMe_3$.

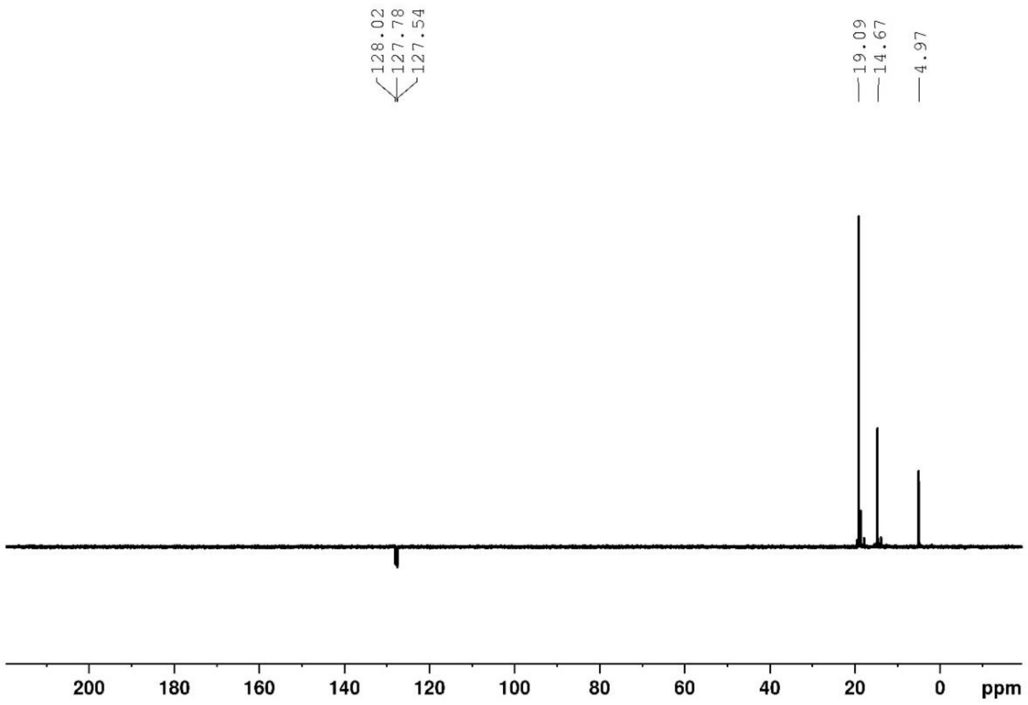

Figure S10. ^{13}C-NMR (125 MHz, 25 °C, C_6D_6) spectrum of *i*Pr_3Si-Se-$SiMe_3$.

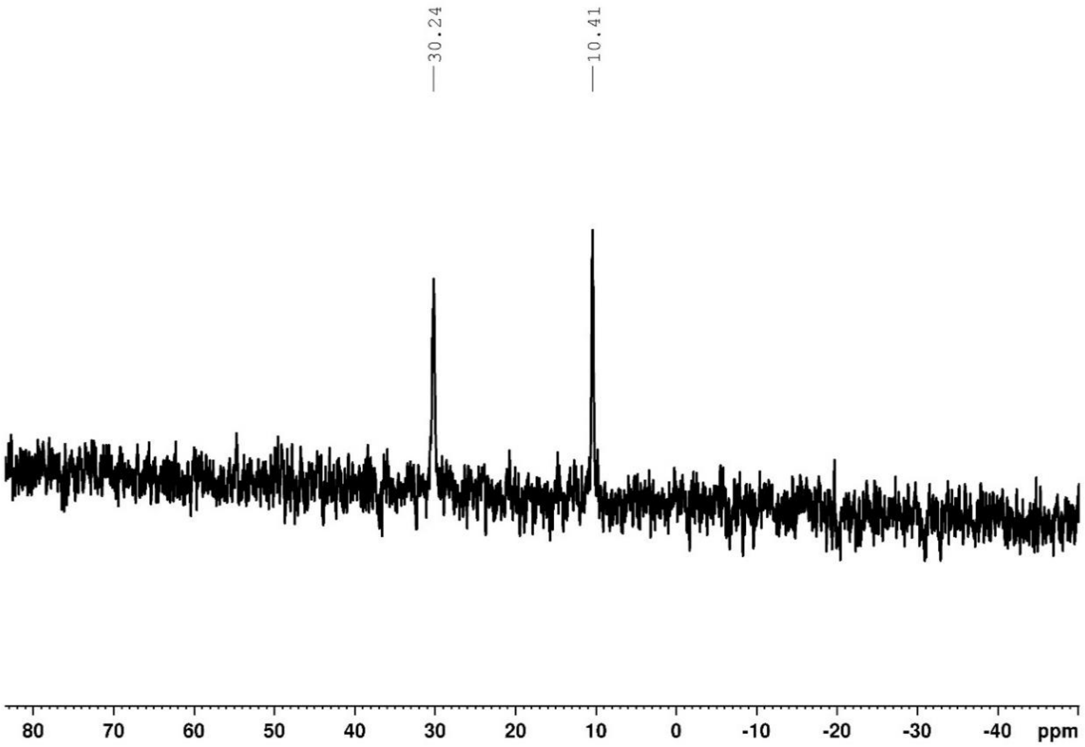

Figure S11. ^{29}Si-NMR (99 MHz, 25 °C, C_6D_6) spectrum of *i*Pr_3Si-Se-$SiMe_3$.

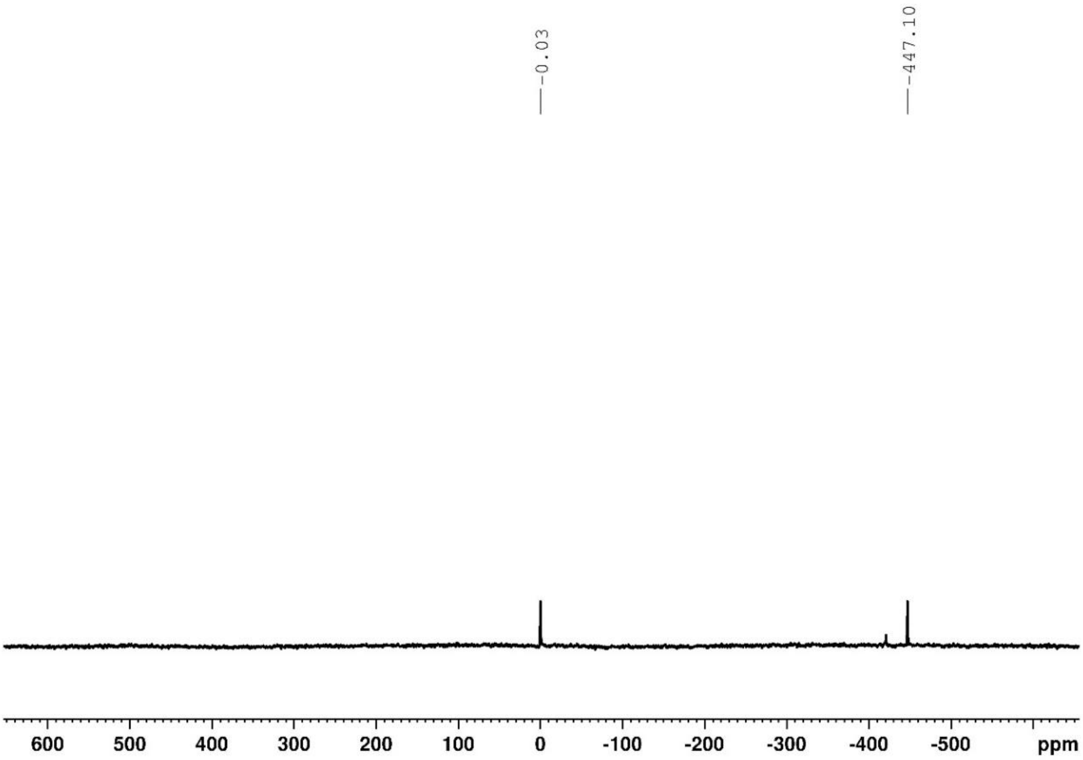

Figure S12. ^{77}Se-NMR (95 MHz, 25 °C, C_6D_6) spectrum of *i*Pr_3Si-Se-$SiMe_3$.

4. GC/MS Records

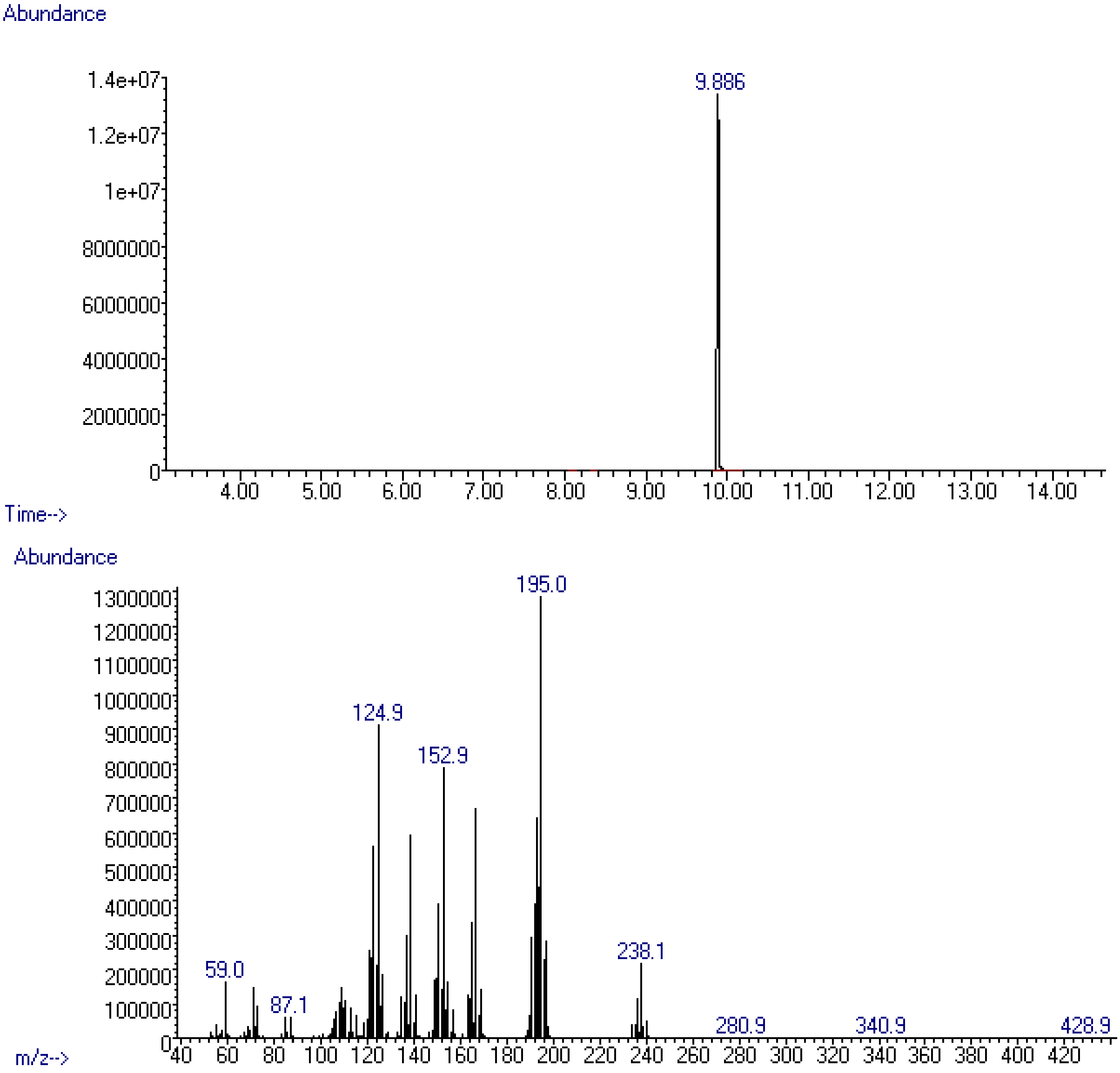

Figure S13. GC/MS record of *i*Pr_3Si-SeH.

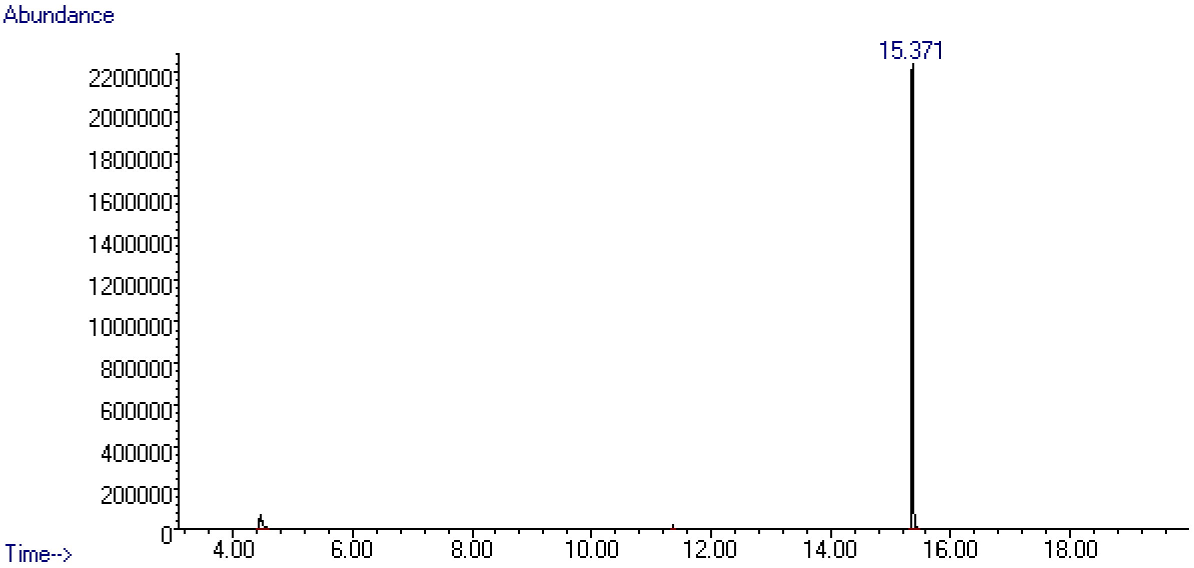

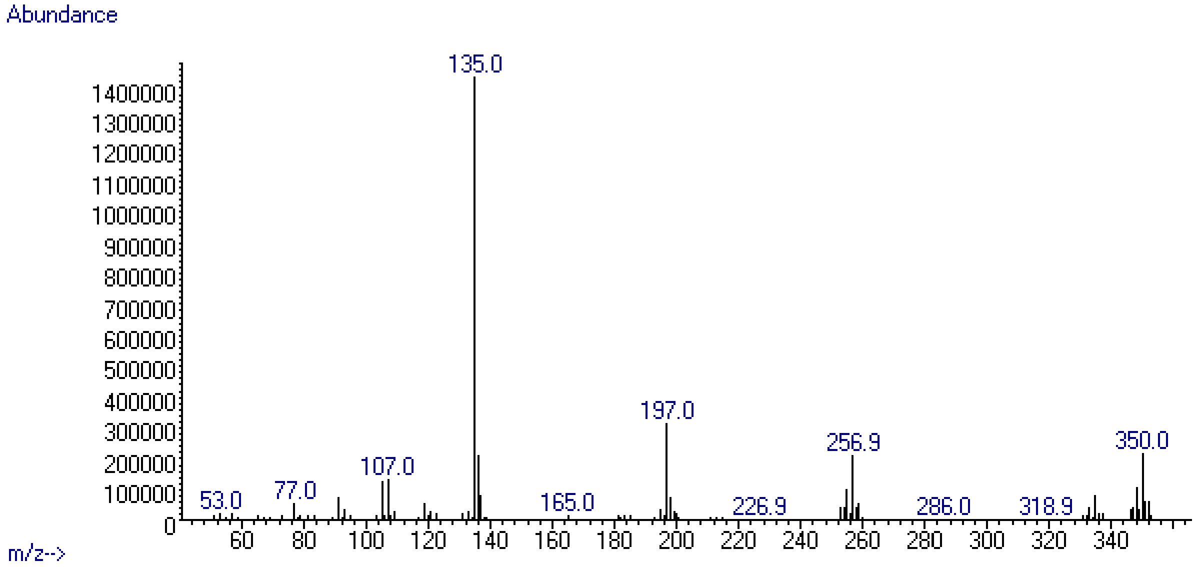

Figure S14. GC/MS record of $PhMe_2Si$-Se-$SiMe_2Ph$.

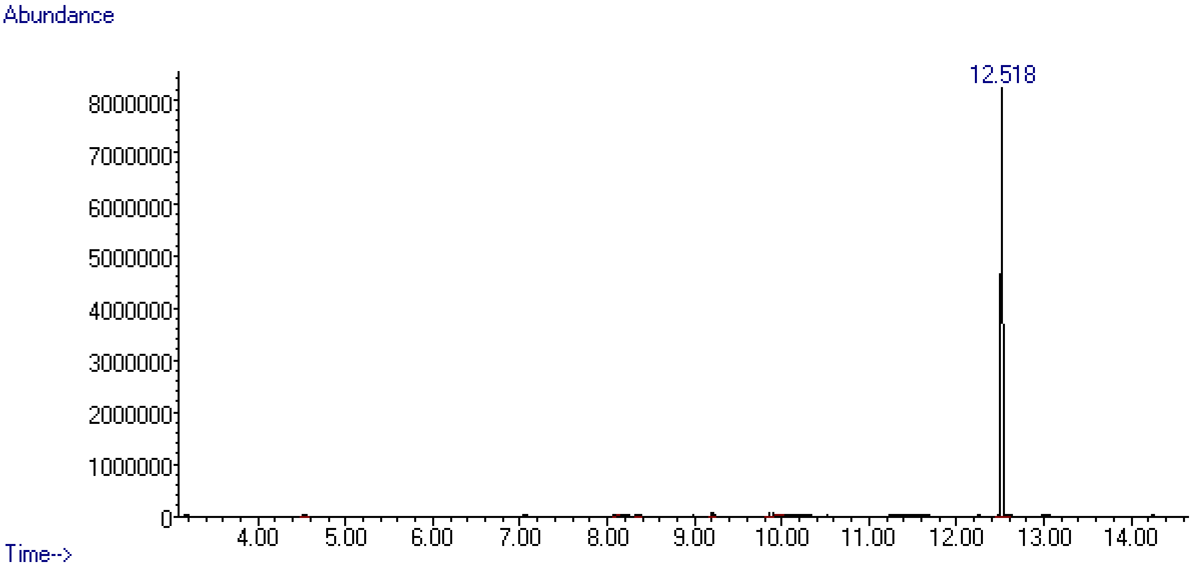

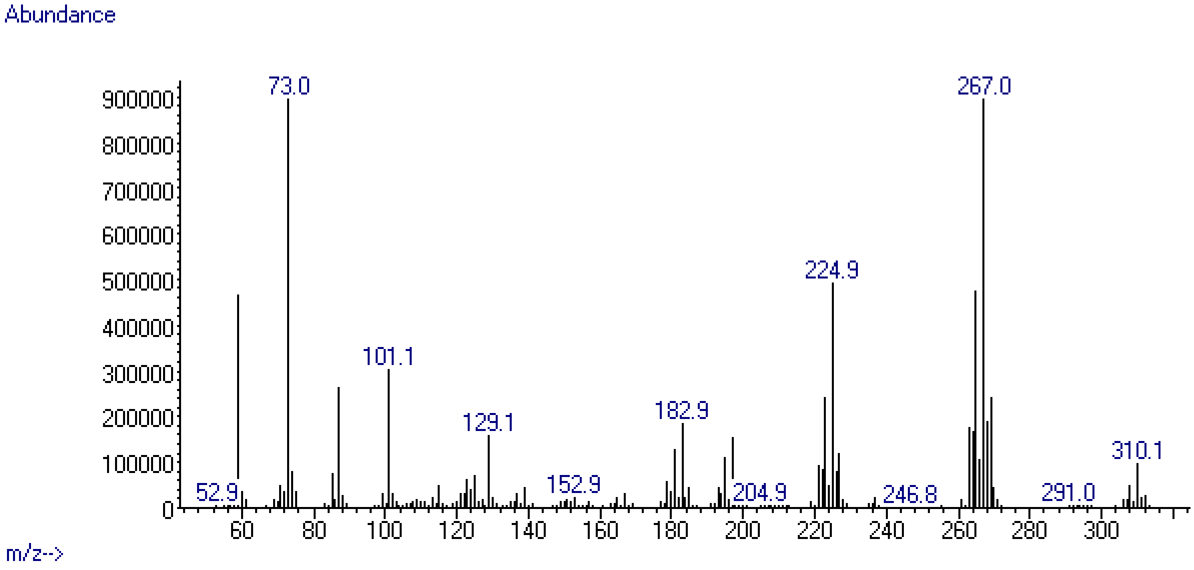

Figure S15. GC/MS record of *i*Pr_3Si-Se-$SiMe_3$.

5. DSC Thermograms

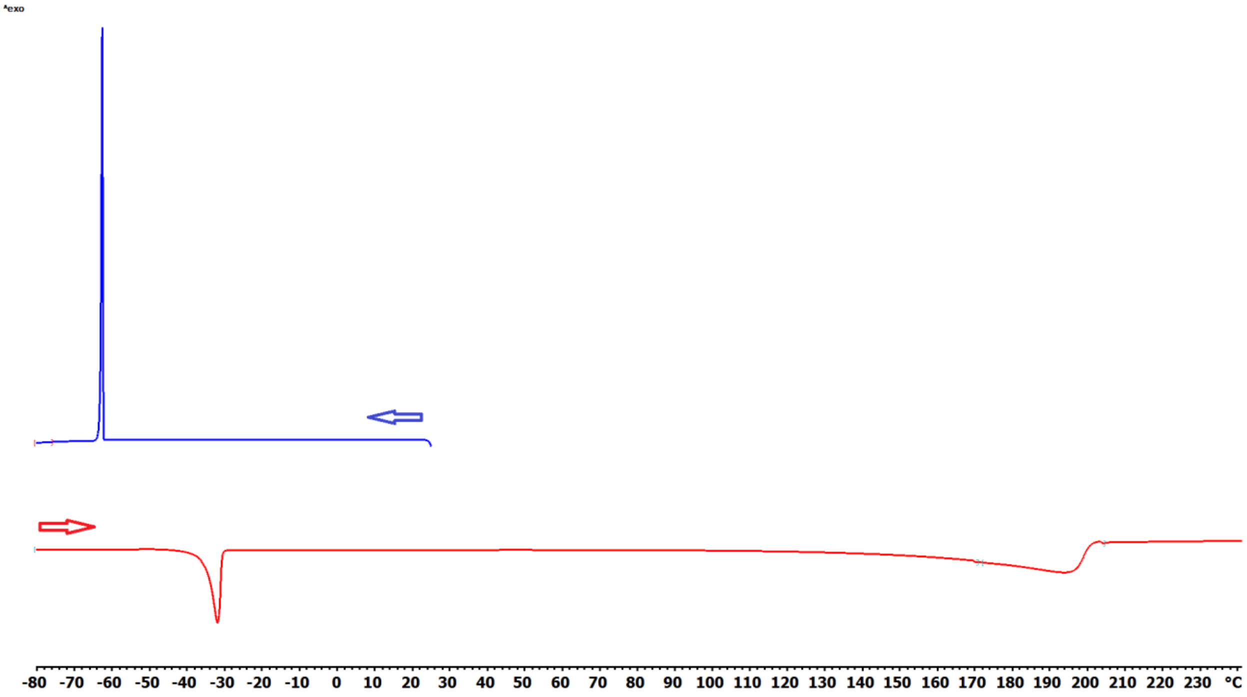

Figure S16. DSC curve of *i*Pr_3Si-SeH.

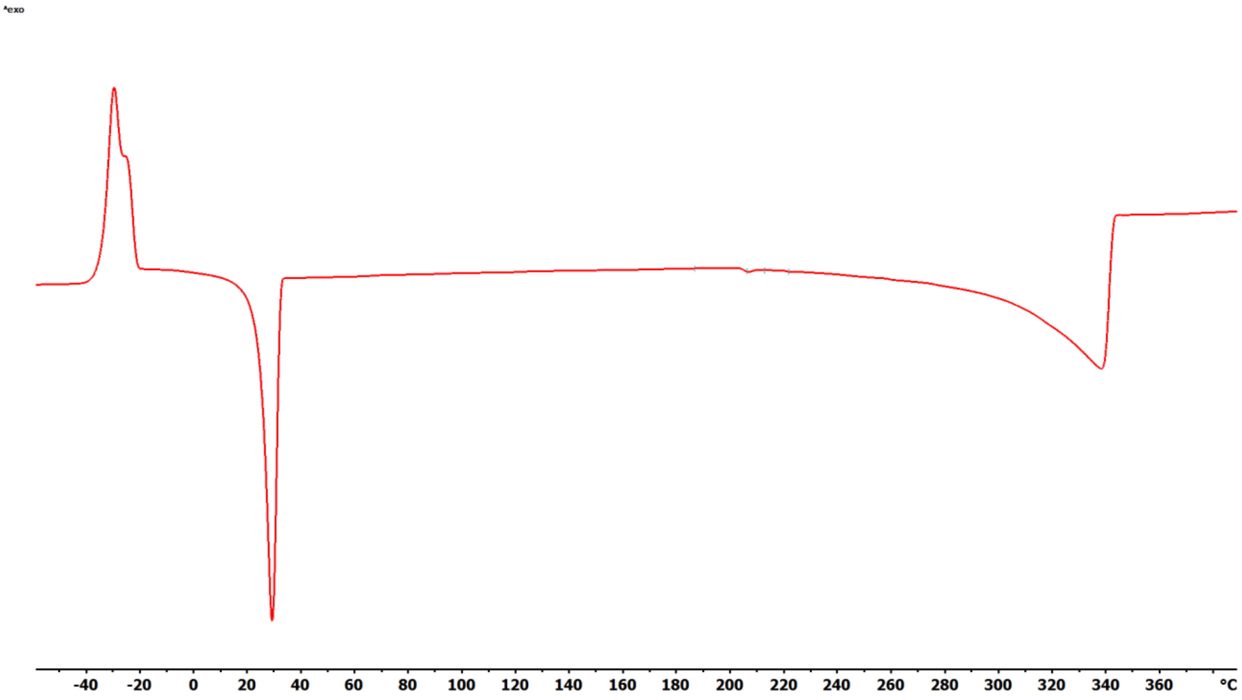

Figure S17. DSC curve of $PhMe_2$Si-Se-$SiMe_2$Ph.

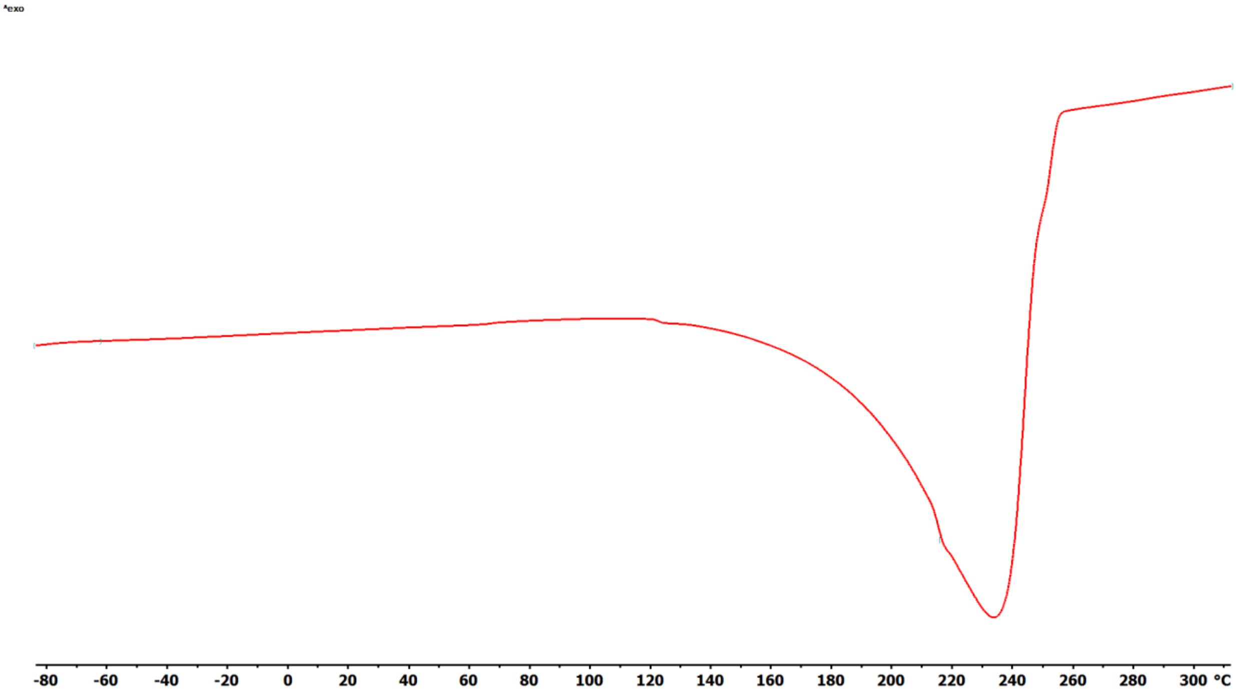

Figure S18. DSC curve of *i*Pr_3Si-Se-$SiMe_3$.

6. TGA

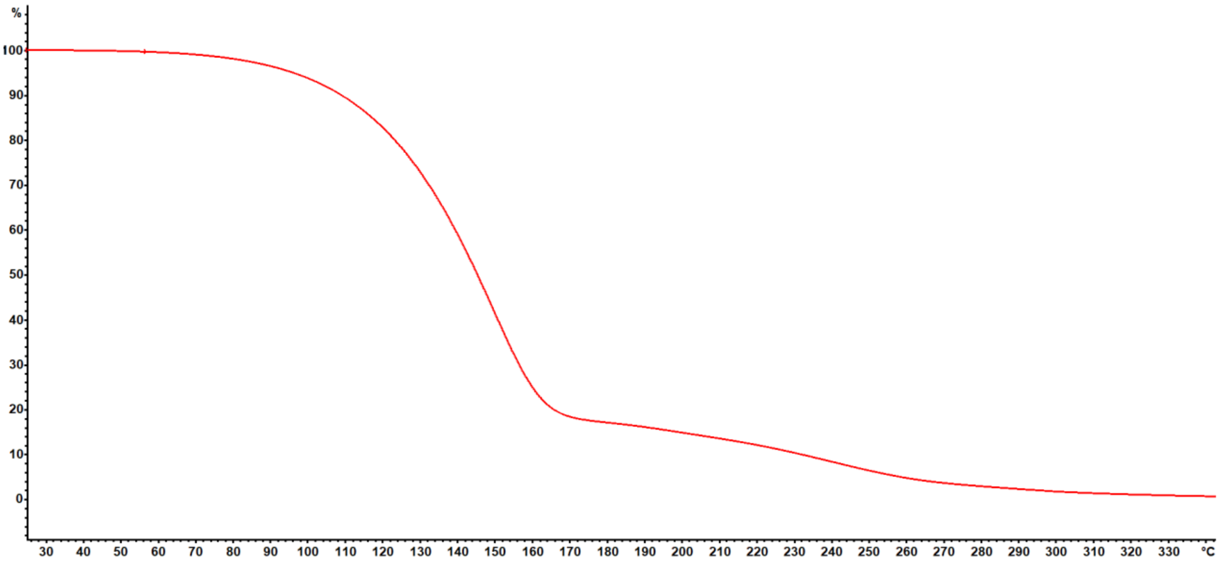

Figure S19. TGA curve of *i*Pr_3Si-SeH.

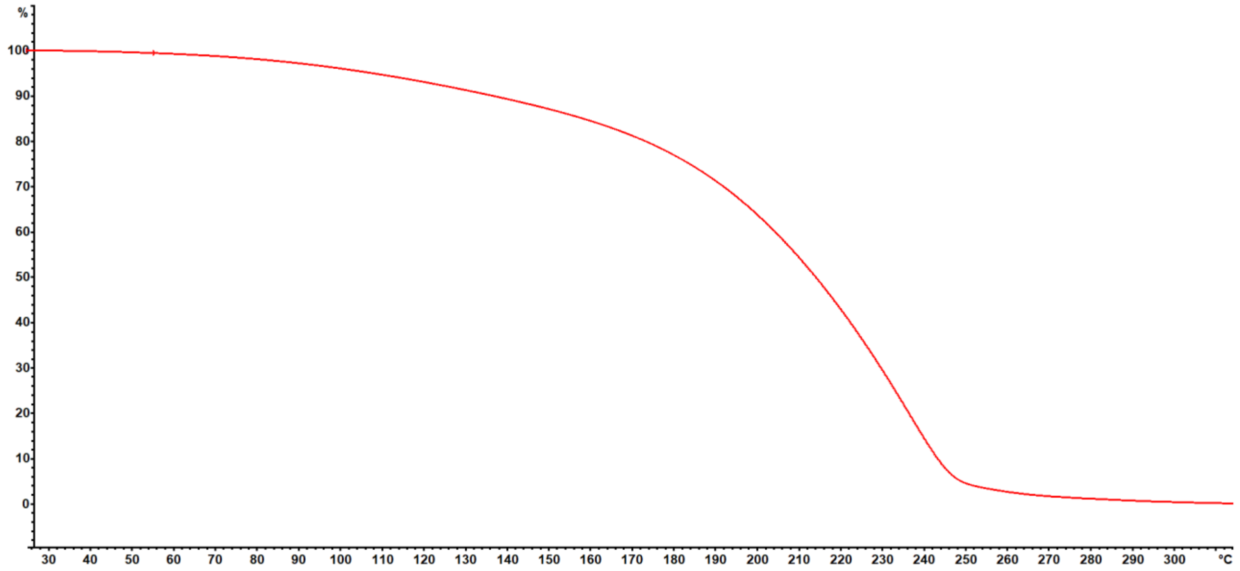

Figure S20. TGA curve of PhMe2Si-Se-SiMe2Ph.

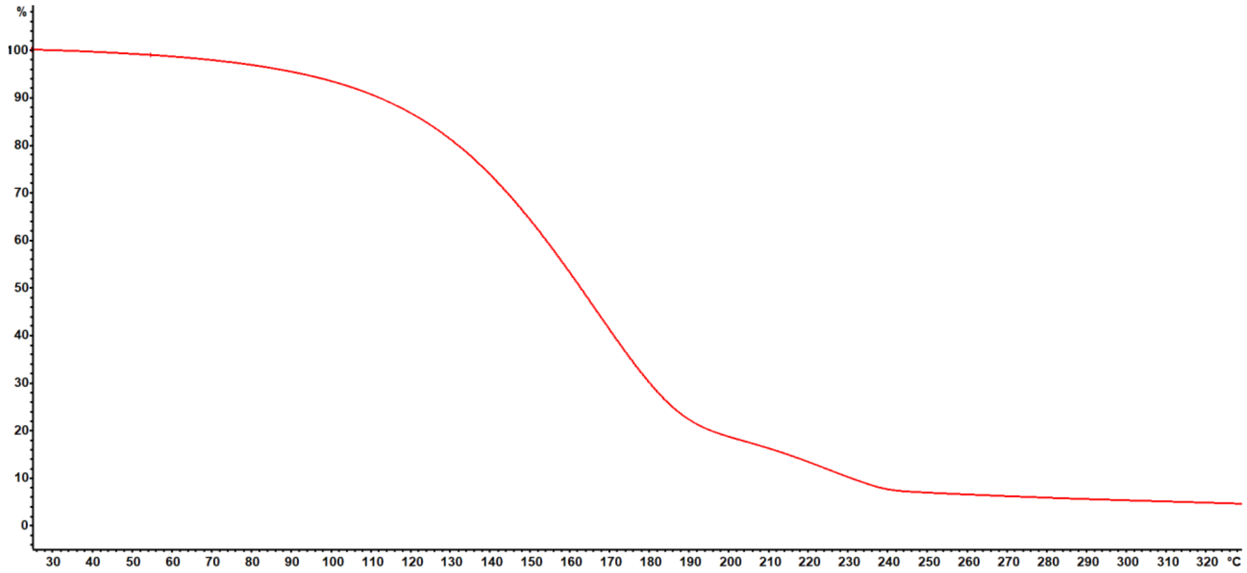

Figure S21. TGA curve of *i*Pr3Si-Se-SiMe3.

Publisher's Note: MDPI stays neutral with regard to jurisdictional claims in published maps and institutional affiliations.

© 2020 by the authors. Submitted for possible open access publication under the terms and conditions of the Creative Commons Attribution (CC BY) license (http://creativecommons.org/licenses/by/4.0/).
